# Blood Lead Level among Children between 8-18 years of Age by Atomic Absorption Spectrophotometry: A Descriptive Cross-sectional Study

**DOI:** 10.31729/jnma.6858

**Published:** 2021-10-31

**Authors:** Yagya Kumari Shrestha, Madhav Prasad Khanal, Shree Krishna Shrestha, Jeewan Shrestha, Navin Shrestha

**Affiliations:** 1 Department of Biochemistry, Nepal Medical College, Kathmandu, Nepal; 2 Department of Surgery, NAMS, Bir Hospital, Kathmandu, Nepal; 3 Department of Dental Surgery, Aarogya Multispeciality Hospital, Madhyapur Thimi, Bhaktapur, Nepal; 4 Emergency Department National Medical College Teaching Hospital, Birgunj, Nepal

**Keywords:** *haemoglobin*, *lead*, *spectrophotometer*

## Abstract

**Introduction:**

Lead is naturally available toxic heavy metal which is one of the main causes of environmental pollution and produces detrimental effects on health, particularly young children. Lead toxicity has become an emerging global burden of disease varying with the age, socio-economic status, occupation, industrialization, social customs and behaviours. The objective of this study was to find out the baseline blood lead level among children between 8-18 years.

**Methods:**

A descriptive study was conducted in Gokarneshwor Municipality, Kathmandu with a total of 160 children between 8-18 years of age from 2018 to 2019 after taking ethical approval from Research and Institutional Review Committee (Reference number: 17-074/075). Informed written consent was taken from the principal along with their respective parents and semi structured questionnaires were asked to students. Sample size was calculated and simple random sampling was done. The data was analyzed using Statistical Package for Social Science version 16. Point estimate at 95% Confidence Interval was calculated along with frequency and descriptive statistics.

**Results:**

The mean blood lead level of the children was 4.39+7.35 μg/dl. Mean hemoglobin being 12.63g/dl; out of 160 children 30 (18.75%) children had blood lead levels. Children with elevated blood lead level had mean haemoglobin level within normal range (13.05gm/dl), however out of 30 children, 8 (27%) had haemoglobin level below normal.

**Conclusions:**

Lead exposure in the children of urban area of Nepal is considerably high as compared to similar studies coducted in similar settings.

## INTRODUCTION

Lead is very toxic cumulative metal and creates health problems.^1,2^ The main sources of lead exposure and its pollution are classified as environmental and occupational.^3^ Children are exposed to lead from sources like lead based paints, pica, contaminated drinking water, toys and lead based peeled, chipped paints especially during renovations of old houses.^4,5^

Center for Disease Control and Prevention (CDC) has recently suggested 5μg/dl as a reference value.^6^ Elevated BLL is often associated with decrease in some trace elements like iron leading iron deficiency anemia affecting synthesis of enzyme δ-amino levulinic acid.^7^

Elevated BLLs also reduce the lifespan of red cells, causing haemolytic anaemia.^8^ Lead toxicity is associated with adverse effects on intellectual development.^9-10^ A study done among older children from urban areas of Chennai has reported prevalence of 52.5%." In Nepal, its distribution and clinical surveys are to be studied in large cohorts.

The objective of this study was to find out the baseline blood lead level among children between 8-18 years.

## METHODS

A descriptive cross-sectional study was carried out on school children aged between 8-18 years residing in Gokarneshwor municipality. Data were collected from 2018 to 2019 after taking ethical approval from the Research and Institutional Review Committee, NMCTH (Reference number: 17-074/075). Children who were willing to participate were included while children suffering from any chronic illness were excluded. Simple random sampling was carried out and the sample size was using the formula:

n = Z^2^ × (σ)^2^ / e^2^

  = (1.96)^2^ × (5.3)^2^ / (1)^2^

  = 108

where,

n = sample size
Z = 1.96 at 95% confidence level
σ = Standard Deviation (5.3) according to a study done among older children from urban areas of Chennai^11^
e = margin of error, 1%.

Adding a non-response rate of 10% the required sample was 119. However, 160 children were included in the study. Total 3ml of blood from each participant was drawn from ante-cubital vein under strict aseptic conditions. Haemoglobin estimation by Drabkin's method in whole blood by using Drabkin reagent (Potassium ferricyanide / Potassium cyanide) at laboratory of Biochemistry Department of Nepal Medical College while BLLs estimation done in serum by Graphite Furnace AAS & Flame AAS applying lysing method at National Academy of Science and Technology (NAST), Lalitpur. Sample preparation for this method was done following Miller et al^12^ procedure i.e. 1+9 dilution with solution composed of 0.2% (NH_4_)_2_HPO_4_, 0.5% Triton X-100 and 0.2% HNO_3_ at Department of Biotechnology, Tribhuvan University, Kirtipur. A semi structured questionnaire was asked from students & values of interest were recorded. The informed written consent was taken. Data was then analyzed using Statistical Package for Social Sciences (SPSS) version 16. Analysis included standard descriptive statistics. Normally distributed variables were expressed in terms of mean±SD (standard deviation), whereas non normally distributed variables were presented as medians and ranges. Continuous data (BLLs) were expressed as mean±SD.

## RESULTS

BLLs were found to be scattered ranging from 0.10-49.56μg/dl with geometric mean of BLL being 4.39±7.35μg/dl, median being 3.00y7g/dl (range: 0.1-49.56μg/dl). Majority of the children were within the age between 14-16 years 37 (42.5%) male and 50 (57.5%) females. Least of children were below 10 years 6 (60%) males and 4 (40%) females. The mean age of children was 13.9±2.18 years. The mean BMI was found to be 20.06±3.77kg/m^2^. Hb was found to be ranged from 6.0-22.80g/dl, mean±SD of Hb being 12.63± 2.91g/dl. (Table 1).

**Table 1:** Study of Age, BMI, Hb and BLL among school children (n=160).

Variables	Range	Mean ± SD	Median
Age	8.00-18.00	13.99±2.18	14.00
BMI (kg/m2)	11.41-33.75	20.06 ± 3.77	19.58
Hb (gm/dl)	6.00-22.80	12.63 ± 2.91	12.60
BLL (μg/dl)	0.10-49.56	4.39 ± 7.35	3.00

Elevated BLL was seen in 14-16 years of age group 15 (9.4%) followed by 11-13 years of age group 8.96 (5.6%) and ≤ 10 years of age 6 (3.75%).

Children with elevated BLL had mean Hb level within normal range (13.05gm/dl), however out of 30 children 8 (27%) had Hb level below normal (Table 2).

**Table 2:** Hb levels among school children (n = 30) with elevated BLL (>5μg/dl).

Haemoglobin (gm/dl)	n (%)
<12	8 (27)
12-13.9	9 (30)
14-15.9	8 (27)
>16	5 (16)

BLL of 105 (65.62%) children was measured by GFAAS method with mean value of 3.72 μg/dl (range 0.10-49.56 μg/dl) while BLL of 55 (34.37%) children was measured by FAAS with mean 5.65 μg/dl (range 1.0-25.0μg/dl) (Table 3).

**Table 3:** BLL by GFAAS and FAAS (n = 160).

BLL	Type	n	Mean ± SD	Range (μg/dl)
	GFAAS	105	3.73±8.18	0.10 - 49.56
	FAAS	55	5.65 ± 5.26	1.0 - 25.0

BLL among samples of20 childrenwere measuredby both GFAAS and FAAS method with mean value of 6.26 μg/dl (range 1.03-11.73μg/dl) and 3.55 vg/dl (range 2.0-4.0μg/dl) respectively. By GFAAS method, a wider range of BLL can be measured than by FAAS method (Table 4).

**Table 4:** BLL by GFAAS and FAAS (n= 20)

BLL	Type	n	Mean±SD	Range (ug/dl)
	GFAAS	20	6.26 ± 3.61	1.03-11.73
	FAAS	20	3.55 ±0.68	2.0-4.0

Among the sociodemographic and environmental variables studied, lead exposure had been related to the age group, frequent consumption of instant noodles, children exposed to renovation work, and children playing with batteries (Table 5).

**Table 5:** BLL and various risk factors (variables 1-8).

Variables	Category	BLL (μg/dl)	
		<5	>5
**Age group**	<10	4	6
	11-13	40	9
	14-16	72	15
	>17	14	0
**Body Mass Index**	Underweight	44	15
	Normal Weight	72	12
	Pre-obese	12	2
	Obese	2	1
**Instant noodles (How frequently child eat instant noodles?)**	Never	4	1
	2-4/month	18	2
	2-3/week	20	12
	>4/week	88	15
Renovation work (Has any renovation work done on your house in the past?)	Yes	75	11
	No	55	19
Type house (type of house a child lives in?)	Traditional (Mud)	55	9
	Modern (Cement)	72	20
	Mix	3	1
Room paint (Are any of the rooms inside the house painted?)	Yes	110	28
	No	20	2
Play dirt (Does a child play in dirt?)	Yes	65	10
	No	65	20
Play batteries (Does a child play with batteries?)	Yes	46	5
	No	84	25

## DISCUSSION

Very high prevalence of the lead exposure has been found in the study conducted by Dhimal, et al.^1^ which was 64.4%, exceeding the cut off points of CDC i.e. ≥5μg/dl. However, in this study so much high exposure is not found but still it is significant (18.75%).

Gautam K et al,^4^ concluded that the prevalence of BLL in children from the industrial city of Nepal was alarmingly high. Children exposed with chipped paints were related to BLL. Though not so high, the significant prevalence of the lead exposure in this study warrants need for screening programmes.

In this current study those children with elevated BLL had mean Hb level within normal range, however 8 out of 30 children had Hb level below normal. Mohan VR et al,^5^ found more than half of the children (57% of 251) were anemic at 15 months of age, and elevated BLLs were not significantly associated with anemia.

Meta-analysis by Wang S and Zhang J^9^ found the mean BLLs of Chinese children living in industrial and urban areas were significantly higher than those of children in suburbs and rural areas. The children's BLLs in China are higher than those of their counterparts in other countries due to its heavy lead pollution. China is considered heavily polluted with lead, especially urban areas. Large scale study is needed in our country to calculate the load of lead pollution.

Etchevers et al.^10^ showed significant association of BLL with the environmental factors such as consumption of tap water in homes with lead service connections, peeling paint or recent renovations in old housing, hand-mouth behavior, passive smoking and having a mother born in a country where lead is often used while in this current study among many environmental factors, only children frequently consuming instant noodles, children playing with batteries, children exposed to recent renovation work at home and age group respectively were related with BLL.

In this study BLL was found to be highly scattered with minimum BLL value, maximum BLL value, mean BLL, median, standard deviation. It may be due to the fact that the subject's BLLs were measured by two methods (FAAS and GFAAS) of different sensitivity. This was found to be lower compared to a similar study done in the urban area of Karachi, conducted by Khan et al.^13^ which showed the mean lead level for the entire group was 7.9μg/dl (SD= 4.5μg/dl). CDC has recommended intervention if a child's BLL is more than 15μg/dl. In this study also those children who had elevated BLL should undergo detailed medical evaluation.

Mitra, et al.^14^ found high BLLs were significantly correlated with low BMI and low Hb status. However, in this current study the mean Hb of the children of the elevated BLL was found to be normal. BMI didn't show relation with BLL.

Lead pollution has created health problems worldwide. Even relatively low levels of exposure can cause serious health conditions including neurological damage. To appear clinical symptoms BLL should be >70μg/dl.^15^ However, detailed clinical history, IQ were not evaluated even those children who had high BLL were not followed up. Sherchand O et al,^16^ had conducted the first cross sectional study in primary school children in Nepal which revealed that the median BLL of school children was 5.5μg/dl. In this study however the level of toxicity was not seen high to appear clinical symptoms.

Ilmiawati et al.^17^ determined BLLs of the Japanese children by Inductively Coupled Plasma Mass Spectrometry (ICP-MS) and found the mean to be 0.96μg/dl and concluded lead exposure was generally low among Japanese children. In contrast, 18.75% children with elevated BLL beyond CDC normal reference range predict that children are alarmingly at high risk of lead poisoning, therefore public awareness and preventive programs are to be recommended.

The present study had few unavoidable limitations. This study could not include children of lower age group due to many difficulties. We had to limit the sample size because of financial limitations. This study could not assess other risk factors including socioeconomic status, residency near industries and did not measure the presence of lead from the other sources of contamination e.g. lead level in soil, paint, dust, water supply, fashion accessories, folk remedies and so on. Also, the study did not investigate cognitive function or intelligence quotient (IQ) in children. As Hb was calculated by Drabkin method by colorimeter accurate Hb measurement could not be done. It warrants the large volume study of lead level measurement in children and its effect in Hb values measured by standard method. Children with elevated BLLs were not followed up or retested. This study did not establish any causal pathway between suspected sources and causes of elevated BLLs. It had a cross-sectional design, and lead exposure throughout the entire childhood development could not be assessed. Other hazardous environmental pollutants (e.g., mercury and cadmium) were not evaluated.

## CONCLUSIONS

Elevated BLLs were more common among school children living in urban area. The GFAAS was a better method to measure BLL than FAAS. Children frequently consuming instant noodles, children playing with batteries, children exposed to recent renovation work at home and the age group related to the elevated BLL. Lead exposure in school children is becoming an alarming health problem in cities of Nepal. Study in large population is recommended for further evaluation of the current situation regarding the lead exposure to children. Screening programmes for BLL at least among school children living in urban areas should be emphasized.
